# Spatiotemporal calcium signaling to unlock the calcium paradox: location, location, location!

**DOI:** 10.1152/ajplung.00262.2026

**Published:** 2026-07-21

**Authors:** Amir A. Zeki, John G. Albeck

**Affiliations:** 1 Division of Pulmonary, Critical Care, and Sleep Medicine, Lung Center, Department of Internal Medicine, University of California, Davis, School of Medicine, Sacramento, California, United States;; 2 Reversible Obstructive Airway Disease (ROAD) Center, University of California, Davis, Sacramento, California, United States;; 3 Department of Internal Medicine, Veterans Affairs Medical Center, Mather, California, United States;; 4 Department of Molecular and Cellular Biology, University of California, Davis, Davis, California, United States

## INTRODUCTION

Signal transduction pathways allow cells to process essential information from their environment and tune their responses appropriately. A long-standing question is how such pathways, composed of receptors, enzymes, and channels, achieve the accuracy and precision needed to support the physiological functions of cells. Evidence accumulated over several decades makes it clear that effective communication requires an interplay of molecular selectivity and spatiotemporal coding (1). This exchange has been carefully studied in a small number of model systems, such as inflammatory signals in macrophages, leading to important insights into how primarily protein-based assemblies encode information (2). However, in many key physiological systems, the mechanisms that discriminate between alternate inputs remain elusive. A case in point is the paradoxical behavior of airway smooth muscle (ASM) cells: how is it that bitter taste receptor (TAS2R) agonists cause ASM relaxation and bronchodilation despite raising intracellular calcium (Ca^2+^), which normally operates to drive contraction? This is indeed a kind of “calcium paradox.” In this journal, Conaway et al. (3) use a suite of modern tools, including deep learning-based computational modeling of ASM ion channel dynamics and single-cell fluorescent microscopy, to bring significant new insight to this question. These tools allowed the authors to detect distinct spatiotemporal calcium dynamics that differentiate TAS2R and histamine inputs and set the tone for an altogether novel and integrative approach in the ASM field.

## KEY FINDINGS

G-protein coupled receptors (GPCRs) play a fundamental role in ASM signaling and cell physiology. Of note, both histamine, a procontractile Gq-coupled GPCR agonist, and TAS2R agonists, such as chloroquine (CQ), activate phospholipase C (PLC) and raise intracellular calcium. However, these stimuli drive opposite outcomes: ASM contraction versus relaxation, respectively. Several key findings reported by the authors are worth highlighting. First, there was a significant difference between the magnitude and synchrony of calcium signals. Histamine caused a synchronized, plasma membrane-localized, rapid, and strong calcium release in ASM cells, nearly twice the magnitude of that produced by CQ. Conversely, TAS2R agonists CQ and FFA (flufenamic acid) caused a lower-magnitude, slower, more variable and asynchronous, and more distributed calcium responses with greater entropy on per-cell analysis. The difference could be due in part to the spatial distribution of the receptors. Although histamine-1 receptors are more uniformly distributed across the cell plasma membrane surface, TAS2Rs may be more localized in specific regions or membrane microdomains; this remains an open and intriguing question. Second, functional outcomes could be explained by distinct ion channel engagements using a computational ion channel model. Histamine-mediated calcium release was consistent with the kinetic profile of voltagegated calcium and sodium channels, leading to membrane depolarization and ASM contraction. By contrast, these models suggest that CQ preferentially engages large-conductance calcium-activated potassium channels (BK_Ca_), small-conductance calcium-activated potassium channels (SK_Ca_), and chloride channels, promoting membrane hyperpolarization and ASM relaxation. This finding adds to the original report by Deshpande et al. (4) that TAS2R-mediated ASM relaxation involves localized calcium signaling at the cell membrane that opens BK_Ca_ channels.

## FINDINGS WITH RESPECT TO CURRENT KNOWLEDGE

The TAS2Rs on human ASM represent a distinct bronchodilatory mechanism different from β_2_-adrenergic receptors. They cause potent ASM relaxation via compartmentalized calcium signaling, BK_Ca_ channel activation, RhoA inhibition, and actin cytoskeleton remodeling, and are resistant to β_2_-agonist tachyphylaxis. Their potent ASM relaxation and bronchodilation is up to threefold greater than β_2_-agonists (4). These receptors also possess pleiotropic properties beyond bronchodilation including anti-proliferation, anti-inflammation, and anti-remodeling, i.e., effects relevant to multiple pathological features of asthma and/or COPD. The work by Conaway et al. (3) complements and expands prior findings from the same authors showing that TAS2R agonists also cause ASM relaxation through distinct mechanisms including inhibition of RhoA GTPase activity and MYPT1 phosphorylation, rather than the typical PKA-dependent pathway utilized by β_2_-agonists (5). Other groups have also demonstrated TAS2R-mediated ASM relaxation via Par3/LIMK/cofilin-dependent actin depolymerization (6). They showed that TAS2R14 activation in human ASM promotes relaxation through cofilin-dependent filamentous (F)-actin severing. Altogether these studies indicate that TAS2R-mediated bronchodilation works via multiple different mechanisms functioning in parallel: contractile apparatus regulation, spatially distinct and compartmentalized calcium signaling, and differential ion channel activation. This pleiotropy in TAS2R molecular signaling is not only intriguing but also makes these receptors a promising therapeutic target for asthma and chronic obstructive pulmonary disease (COPD).

## NEW INSIGHTS

As a cationic secondary messenger calcium can manifest diametrically opposite cellular responses, i.e., ASM contraction versus relaxation (“calcium paradox”). In the work accomplished by Conaway et al. (7), this paradox may be elucidated through differences in calcium’s spatiotemporal signature. Furthermore, this does not necessarily occur via other secondary messengers or different downstream effectors as far as we know. This challenges a foundational assumption in cell signaling biology and reveals the power of using approaches that allow detailed microscopic quantifications and real-time spatiotemporal measurements (7). The findings herein suggest that calcium is an “information-rich” signal by way of its spatiotemporal activity, rather than a binary switch representing procontractility versus prorelaxation. The intracellular location and energetic context, as well as plasma membrane receptor locations and density likely matter as well.

Although still speculative to some degree, calcium spatial compartmentalization may be an important aspect of this response. It is thought that TAS2R agonists drive calcium changes toward the periphery of the cell close to the plasma membrane, which activates hyperpolarizing potassium channels. By contrast, histamine induces a global intracellular calcium surge that causes ASM contraction. This peripheral versus global distribution of calcium may function as a “decision boundary,” where calcium at the membrane periphery activates hyperpolarizing potassium channels leading to relaxation, whereas calcium that emanates into deeper cytosolic spaces activates the calmodulin-myosin light chain kinase (MLCK) contractile machinery (8).

Another key insight has to do with the chaotic nature of the TAS2R-agonist-induced calcium signaling dynamics. The use of information-theoretic entropy is established in the signaling field, but its use to characterize TAS2R-mediated calcium signaling in ASM is novel. The data suggest that high entropy (i.e., variable, asynchronous) calcium signals stimulated by TAS2R agonists are read differently by the ASM cell than low entropy (i.e., synchronized, coherent) signals from histamine stimulation. This occurs even though both involve key aspects of the PLC/IP_3_ signaling cascade. This concept seems to parallel emerging ideas in vascular smooth muscle biology, where oscillatory versus sustained calcium patterns activate different transcription factors (9, 10).

The broader implications of this work involve the concept of “calcium encoding” from the field of calcium biology, i.e., that calcium functions as a frequency- and amplitude-modulated signal in ASM. The cell does not simply ask “is my calcium up or down?” Rather, the cell asks, “how much, is it synchronized, for how long, and where?” Indeed, calcium is not a mere on/off switch but rather is an information-laden cation capable of encoding multiple and possibly opposing instructions simultaneously. Furthermore, this calcium story hints that other apparent paradoxes in GPCR signaling, where the same second messenger produces different outcomes depending on the receptor, may be resolved via careful spatiotemporal analysis of signaling dynamics rather than simply invoking the presumed engagement of separate downstream pathways (11).

## LIMITATIONS

The authors acknowledge that their method has known limitations worth mentioning. Although image segmentation algorithms have made major advances through deep learning, there is still the potential for motion artifacts, varied cell morphology, and overlapping cells, each of which can limit accuracy. The ordinary differential equation (ODE) frame-work is also inherently sensitive to noise in calcium transients and the potential for incorrect kinetic parameter assumptions, which could introduce variability in the model’s predictions. The authors correctly point out that technical limitations have prevented efforts to assess TAS2R plasma membrane distribution. It is possible that differential localization of this family of GPCRs may contribute to the distinct calcium kinetics observed. It remains an open question as to how (and whether) the spatial distribution of receptors at the ASM plasma membrane impacts intracellular signaling compartmentalization. Whether TAS2R’s orthosteric binding pocket occupied by endogenous cholesterol has anything to do with its membrane location, interactions, or relationship to lipid rafts is another valid question (12). Although the TAS2R agonist CQ is pleiotropic and could have influenced the observed calcium responses via off-target effects, the authors also used FFA which is known to activate only one subtype of TAS2Rs, the TAS2R14. Yet, despite these efforts, much work remains regarding the development of highly specific TAS2R agonists with the efficacy needed to impact airway function.

## REMAINING UNANSWERED QUESTIONS

Sorting out pleiotropic from direct effects of activated TAS2Rs on calcium and ASM function is an active area of investigation. As the authors correctly point out, TAS2R pharmacology knowledge is still limited including these receptors’ biophysical structures, pool of available ligands, and the large number of other TAS2R subtypes. Still, the approach and analytical tools utilized in this study could be used in future investigations to identify better TAS2R agonists that could be further developed as novel bronchodilators. The main challenge with clinical translation is the development of selective, potent, and safe agonists for human use. Gaining deeper insights into the most complex problems of biology will increasingly require the use of high-fidelity computational approaches and the incorporation of spatiotemporal methods at both single-cell and living tissue levels.
